# Research Progress on Nanomaterials for Tissue Engineering in Oral Diseases

**DOI:** 10.3390/jfb14080404

**Published:** 2023-08-01

**Authors:** Tong Jiang, Wen Su, Yan Li, Mingyuan Jiang, Yonghong Zhang, Cory J. Xian, Yuankun Zhai

**Affiliations:** 1 School of Stomatology, Henan University, Kaifeng 475000, China; tongjiang@henu.edu.cn (T.J.);; 2 Kaifeng Key Laboratory of Periodontal Tissue Engineering, Kaifeng 475000, China; 3 Department of Pharmacy, Huaihe Hospital, Henan University, Kaifeng 475000, China; 4 Department of Orthopaedics, The 2nd Hospital of Shanxi Medical University, Taiyuan 030001, China; 5 UniSA Clinical and Health Sciences, University of South Australia, Adelaide, SA 5001, Australia

**Keywords:** oral nanomaterials, non-viral vector system, oral disease therapy, tissue engineering applications

## Abstract

Due to their superior antibacterial properties, biocompatibility and high conductivity, nanomaterials have shown a broad prospect in the biomedical field and have been widely used in the prevention and treatment of oral diseases. Also due to their small particle sizes and biodegradability, nanomaterials can provide solutions for tissue engineering, especially for oral tissue rehabilitation and regeneration. At present, research on nanomaterials in the field of dentistry focuses on the biological effects of various types of nanomaterials on different oral diseases and tissue engineering applications. In the current review, we have summarized the biological effects of nanoparticles on oral diseases, their potential action mechanisms and influencing factors. We have focused on the opportunities and challenges to various nanomaterial therapy strategies, with specific emphasis on overcoming the challenges through the development of biocompatible and smart nanomaterials. This review will provide references for potential clinical applications of novel nanomaterials in the field of oral medicine for the prevention, diagnosis and treatment of oral diseases.

## 1. The Clinical Demand, Development and Delivery Challenges of Nanomaterials for Oral Medicine

Oral, maxillofacial and oropharyngeal tissues carry out important physiological functions such as chewing, swallowing, speech, appearance and sensation. However, the oral cavity and oropharynx have complex internal and external environments, which are vulnerable to physical, chemical and microbial risk factors [1]. In addition, the oral cavity and oropharynx are also prone to become common pathological and cancerous sites. The mouth, as the entrance of the body, connects the external and internal environment. The oral ecosystem, consisting of three main parts (namely oral tissue, saliva and oral microbes) and constantly having dynamic changes, is frequently exposed to food, drugs, saliva, microorganisms and other endogenous and exogenous situations [2,3,4]; thus, its hemostasis can be affected by a variety of factors. Oral and oropharyngeal diseases, which have high natural prevalence and can significantly damage the physical and mental health of people around the world, include oral and oropharyngeal cancers, oral mucosal disease, dental caries, periapical inflammation, pulpitis, periodontal disease, implant infection and others [5,6]. Oral diseases may also become a source of infection and induce diseases of other physiological systems [7]. According to epidemiological studies, the prevalence of oral cancer is generally high (ranking sixth in malignant tumors worldwide) [8]. Periodontitis is the most common chronic oral disease with a prevalence rate of over 90%, which is an important cause of tooth loss [9,10]. Periodontal tissue destruction leads to alveolar bone resorption, which can result in tooth loosening/shedding and dentition defect/loss and impair multiple functions such as chewing, pronunciation and aesthetics. Furthermore, facial trauma, tumor removal, tooth extraction and developmental abnormalities may lead to non-healing oral and maxillofacial defects [11].

The available data suggest that, according to the latest definition of oral health by the World Health Organization (WHO), the number of oral diseases in the world’s population exceeds 3.5 billion [12], including 2.3 billion having untreated caries in permanent teeth, 796 million having severe periodontitis and 267 million having total tooth loss. For these individuals, the financial burden and social/psychological pressure are increasing. Based on incomplete statistics, the global sales of titanium dental implants have reached AU$6.3 billion in 2021 and are expected to reach AU$8.4 billion in 2028 [13]. Thus, oral diseases have become a global health problem which need urgent attention and solutions [14].

Many oral diseases can seriously affect the local oral pathological microenvironment, and the effectiveness of traditional local drug therapy is known to be impaired by flowing saliva. For the systemic delivery of therapies, biological safety, cell targeting, transfection efficiency, nanomaterial accumulation concentration and side effects are issues that need to be considered. Encouragingly, however, over the last decade, great progress has been made in development and new delivery strategies for advanced materials to overcome obstacles for the treatment of oral diseases. So far, treatment of almost all oral diseases depends on the development and promotion of materials science and technology, and the booming development of advanced materials and nanotechnology simultaneously promotes the innovation of oral disease treatment methods and strategies. A range of novel advanced biological nanomaterials, including metal nanoparticles, polymers, hydrogels, nano-films and nanofibers, holds great potential as stem cell and drug carriers for drug delivery and tissue engineering scaffolds [15]. Whether it is prevention, treatment, antibacterial, anti-inflammatory or pulp regeneration and denture selection, nanomaterials have demonstrated their powerful functions in stomatology with some unique advantages [16]. More professional and more functional dental nanomaterials have been applied broadly.

**Figure 1:**
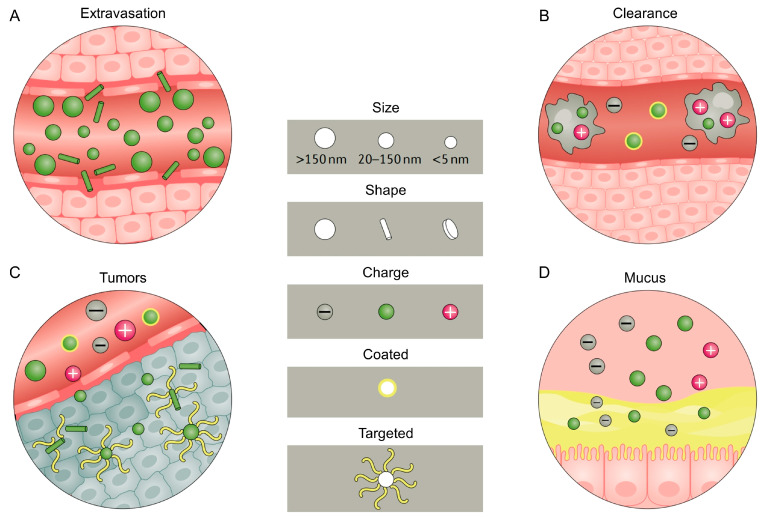
Physicochemical properties of nanoparticles impact their distribution. Sizes, positive and negative charges, shapes, surface coating and modification are factors that determine the performance of nanoparticles in the circulatory system. (**A**) Spherical and large-sized particles are easier to be adsorbed by tissue walls and cells in the circulatory system, while rod-shaped nanoparticles can translocate from within blood vessels into tissues or cells by exosomes. (**B**) Nanoparticles with bare surfaces or more positive charges are eliminated more quickly by the reticuloendothelial system. (**C**) Rod-shaped, targeted and neutral particles exhibit features of better tumor tissue permeability, (**D**) while smaller, positively charged modified or surface-coated nanoparticles have better biological and mucosal barrier penetration. Reproduced with the permission, copyright Springer Nature, 2021.

Limited by the complex microenvironment of the oral cavity, nanomaterials for the treatment of oral diseases either via systemic or via local administration are facing the challenges of biological, physical and transmembrane barriers [17] (Figure 1). Firstly, it is not easy to achieve acceptable nanomaterials delivery and biodistribution efficiency even under routine physiological conditions in humans. Nanomaterials entering the circulatory system first face physical and biological barriers, including rapid clearance due to enzymatic degradation, shear force, cell phagocytosis and protein adsorption. It is not feasible to perform systemic administration, as it greatly reduces the concentration of nanomedicine reaching the target lesion site [18]. In addition, the clearance of the nanomedicine due to the biological barriers can vary, not only with different types of oral diseases, but also with individual differences in patients’ physical conditions of the circulatory system and oral microenvironments, the differences in which pose difficulty for characterization and challenges in the nanomedicine’s application universality.

The stability, drug delivery effect and tissue engineering application of nanomaterials are influenced by the blood circulation system, lymphatic system, excretory system, immune phagocytosis system and other factors. While the physical and chemical properties of biomaterials determine the special effects of various internal and external environmental factors, nanomaterials can be easily removed from the circulation through the interaction of the mononuclear phagocytic system and the reticuloendothelial system [19]. Tissue engineering materials can produce immune responses such as inflammation due to their physical and chemical properties, and these responses are sensitively regulated by phagocytes (macrophages), monocytes (leukocytes) and dendritic cells [18,20]. Nanomaterials are recognized by serum immune active factors, interact with serum proteins, stimulate the innate immune system to enable the particles to be engulfed by mononuclear macrophages and consequently cause inflammation and other related immune responses [21]. The human reticuloendothelial system, including the liver and spleen, can quickly identify and remove exogenous substances in the blood, ingest nanomaterials and accumulate the particles in the spleen and liver [18,20]. Kidney filtration also poses new challenges to the application of nanomaterials [22]. For instance, systemically administered nucleic acid drugs are easily excreted by the kidneys through glomerular filtration and are excreted into urine within one hour [23].

Penetration is the key process for nanomaterials to cross the circulatory system to reach the target tissue for biological distribution [24]. Cell–tissue penetration depends on the comprehensive properties of nanomaterials (including size, charge, hardness and functional group modification), as different sizes of nanoparticles (NPs) can lead to different organ distributions [25]. NPs face new considerable challenges in tissue targeting and cell uptake after reaching the target site (e.g., lesion site) through crossing biological and physical barriers. The cell membrane is a natural barrier to prevent exogenous nanomaterials from entering cells, and the ability of NPs to cross the phospholipid bilayer affects their endocytosis into cells. In addition, the endosomal escape ability also determines the intracellular release efficiency of nanomaterials.

All the above challenges and obstacles together have severely limited the application of nanomaterials in the treatment of oral diseases, and thus, further work is required for the development of new nanomaterials and novel delivery methods. Designing new nanomaterials and developing their appropriate delivery systems that can overcome the aforementioned delivery barriers simultaneously will be a big challenge. To achieve effective drug release and tissue engineering applications, they should meet the following requirements: (i) favorable biocompatibility, degr adability and non-immunogenicity; (ii) avoidance of rapid clearance by the liver and kidney and uptake by the endothelial reticular system; and (iii) avoidance of the encapsulation by endosomes and the degradation by lysosomes in target cells.

## 2. Nanotechnology Platforms

**Figure 2:**
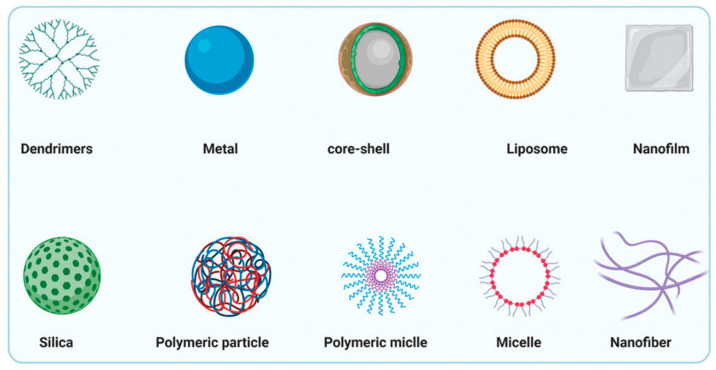
Schematic illustration of nanomaterial delivery systems for oral disease treatment and tissue engineering applications. Each feature example of nanoparticles has multiple subclasses. Non-viral vector systems include polymeric systems (such as micelle, nanogel, linear chain, dendrimer and polymersome), inorganic systems (such as Au/Ag, quantum dots and silica NPs), liposomal and exosome systems, nucleic acid agent-based nanostructures and compounded nanomaterials that may possess some advantageous functions. Nanomaterials have their own strengths and disadvantages in terms of freight, delivery and disease responsiveness. Reproduced with the permission, copyright John Wiley and Sons, 2021.

Advances in biological nanotechnology and materials science have brought great hope to overcome the aforementioned challenges. Nanotechnologies are considered to be the most promising way to prepare novel nano-scale biomaterials (nanomaterials) that can cross biological barriers, and the preparation of nano-formulations can be through electrostatic interactions, trapping or covalent binding [26,27]. So far, different types of nanomaterials have been extensively developed as delivery vehicles and tissue engineering materials [28], and various biological nanomaterials have emerged (Figure 2). Nanoparticles can have the following structural advantages. They can be customized for drug loading and protection. Their sizes, shapes and surfaces can have excellent design properties, which are conducive to increasing the half-life in the blood circulation. In addition, nanomaterials can be easily modified (e.g., by using specific ligands) for precise biofunctionalization to exert their targeting functions.

Nanomaterials have specific properties, such as adjustable size, large specific surface area and easy surface modification, which can enable them to effectively pass through blood vessels and tissues and to accumulate efficiently at the lesion site. With the improvements in transfection efficiency, specificity and safety, the number of biomedical materials in clinical trials has gradually increased [29]. Inorganic nanomaterials, including gold, silver, platinum nanoparticles, quantum dots, graphene, magnetic nanoparticles and silica, are widely used in nano-drug delivery and tissue engineering applications. Quantum dots are semiconductor inorganic crystals with excellent photostability and biological activity [30]. Gold nanoparticles (AuNPs) are used as a promising gene drug delivery carrier due to their excellent biocompatibility, easy synthesis, high specific surface area and easy functional modification [31]. Organic nanoparticles include cationic liposomes, polymer vesicle/micelle, cationic polymer nanoparticles and nucleic acid nanostructures. Drugs can be combined within nanoparticles through chemical bonding synthesis and physical interaction [32]. Local drug delivery devices such as nanofibers, polymer micelles, liposomes, hydrogels, nano-films and oral patches can significantly affect the efficacy and safety of drugs. Other materials such as biocompatible materials and ligand-mediated targeted carriers have research potential to a certain extent.

Single-function nanomaterials may have difficulty in dealing with the complex diseases caused by multiple pathogenic factors in intractable cases. Under the structural advantages, nanomaterials have excellent design properties, of which researchers have devoted great efforts to developing novel and smart agents for synergistic therapeutic effects. For example, during the nanomaterial delivery process, vectors are required to keep stable in the circulation, materials utilize acidic microenvironments or cell surface receptors to achieve selective targeting effects, optimize biological distribution and reduce off-target toxicity, while rapid disassembly is also necessary for drug release after target cells uptake to maximize materials functionality and minimize side effects along with the synergistic therapeutic effects [33]. However, nanomaterials still have substantive problems to overcome, such as cytotoxicity, transfection efficiency and cell targeting. The ultimate goal is to prepare non-toxic, efficient, low-immunogenic and targeted bio-nanomaterials. Therefore, more research is still needed to find better carriers, which will provide more powerful help for the development of biomedical engineering.

## 3. Nanomaterials in Prevention of Oral Diseases

Nanomaterials can be used for oral health care. Modern medicine for oral health has become more intensive for preventing microbial-related diseases, as nanomaterial technology can block and/or partially reverse the progression of oral diseases. By changing the oral microbial environment, the healthy oral microecological balance can be promoted to prevent the occurrence of oral diseases. For example, nano-silicon particles can increase the amount of minerals in the mouth and prevent tooth decay, and nanometer calcium particles can promote remineralization of teeth and improve their resistance to corrosion.

### 3.1. Prevention of Bacterial Growth in the Mouth

The diameter of dental antibacterial biomaterials is usually between 1 and 200 nm. Their large specific surface area and high charge density can provide favorable conditions for their interaction with negatively charged surfaces of bacterial cells and enhance the antibacterial activity of nanomaterials. Thus, they have a broad application prospect in antibacterial therapies for oral diseases [34,35], which, currently, is one of the main directions of dental nanomaterials developed.

Some nanoparticles, such as zinc oxide, Au/Ag and polyethylene, have been chosen to be added into toothpaste or adhesions to inhibit bacterial growth. The main mechanisms of action include their ability of destroying bacterial membranes, interfering with transmembrane electrical transport, inhibiting sugar metabolism, generating reactive oxygen species, replacing magnesium ions required for oral biofilm enzyme activity and blocking DNA replication [36,37,38,39]. Recent work has demonstrated that antibacterial nanostructure coating has the function of effectively inhibiting bacterial adhesion and can contribute to bacteria sterilization while maintaining the integrity of saliva biofilm, and the antibacterial treatment effect of nanoparticles has been shown to depend on the particle size to some extent [40]. Long-chain polymer chitosan has been included as a potential antibacterial agent for dental applications [41]. When the pH is <6.5, protonation of chitosan amino groups occurs, and the positively charged chitosan can lead to the increase in membrane permeability as well as the increase in ion and protein outflow [42]. The chitosan can bind to the DNA of *Staphylococcus aureus*, *Escherichia coli* and other microorganisms to inhibit their mRNA transcription and affect the protein translation process.

### 3.2. Prevention of Demineralization and Promotion of Hard Tissue Mineralization to Control Caries

Inhibition of demineralization of hard tissue contributes to effective prevention and control of the progression of caries. Nano calcium phosphate fillers bind to demineralized enamel or dentin to form a protective film which can efficaciously keep the destruction of plaque acids within limits. Calcium carbonate biomimetic nanomembranes have advantageous abilities of tissue adhesion and releasing high concentrations of calcium ions slowly, while at the same time inducing the increase in the microenvironment pH range and thus effectively promoting the remineralization of the damaged tooth enamel in the early stage. Fluoride has a certain inhibitory effect on bacteria and enzymes and can be used for caries prevention by reducing the acid production capacity of bacteria. Nano-scale calcium fluoride (CaF_2_) can be modified to substitute the hydroxyl of hydroxyapatite for fluorapatite preparation, which can participate in the lattice structure formation of tooth enamel, forming the protective layer of fluorapatite and creating an effective improvement for increasing the strength and acid resistance of teeth.

Furthermore, nanoparticles added into the toothpaste are evenly distributed, and the uniform dispersion has been shown to be beneficial in improving the ability to clean teeth and preventing the formation of calculus dentalis [43]. Doxycycline nanogel has also achieved some success in preventing alveolar bone absence [44,45]. Zhou et al. [46] synthesized the NACP (nanoparticles of amorphous calcium phosphate) composite containing DMAHDM, which has shown good antibacterial properties as it can inhibit the growth and metabolism of *Streptococcus mutans*, *Lactobacillus acidophilus* and *Candida albicans*. In addition, it can release calcium and phosphorus ions, promote remineralization and effectively protect the dentin.

Since the resin bonding interface is prone to the formation of microleakage between the filling material and the tooth due to the long-term exposure to the oral environment, the entrance and presence of residual microorganisms in the dentin may lead to secondary caries. Melo et al. [47] added nano-silver particles and NACP into the adhesive to study the bonding strength of dentin and its influence on dental plaque biofilm. The modified adhesive was shown to significantly reduce the activity and acid production capacity of dental plaque biofilm. Thus, dental repair materials with special nanomaterials can prevent cavities and protect teeth from microbes. Antibacterial nanomaterials can be used not only as fillers, but also in other stomatological materials to prolong the service life of implants.

## 4. Diagnosis of Oral Diseases

Early diagnosis represents the goal of modern medicine and plays an important role in prognosis and further treatment. Many kinds of novel biomarkers for oral disease diagnosis, including microbiomes, cell metabolites, proteins and nucleic acids, have been developed and used widely in basic research and gradually in clinical applications. They are believed to be helpful in elucidating the development process of oral diseases. The complex anatomy of the mouth, the presence of saliva and other physical and chemical characteristics of the environment, such as pH, oxygen content and temperature, determine the organisms that colonize the oral microecosystem [48]. The oral ecosystem consists of unnumbered highly differentiated microorganisms, including bacteria, fungi, protozoa, mycoplasma and viruses. Oral microorganisms are closely associated with the occurrence and deterioration of dental caries/plaque, periodontitis and general diseases, especially causing secondary infections [49,50,51]. For example, saliva and gingival crevicular fluid (GCF), which contain peptides, proteins, enzymes, mucins, cytokines and electrolytes, are habitats of shed cells and microorganisms, which are stimulated and controlled by sympathetic nerves and pathological factors [52]. Fluctuations in internal or external microenvironmental elements can impact biological balance, leading to salivary gland dysfunction, oral diseases and even systemic physiological/pathological disorders of organisms [34,53]. Oral health can be measured by assessing saliva metabolism, and the monitoring of the oral microecosystem not only helps to maintain oral health, but also has a potential impact on overall health [54].

Despite its promising prospect, the monitoring of oral ecosystems is still limited due to the dynamic changes in the oral ecological environment and the shortage of advanced detection techniques with clinical application potential. With the emergence of nanotechnology, biomarker detection has developed rapidly for disease diagnosis and intervention [55,56]. Combined with advanced manufacturing technology, nanomaterials which are easy to be modified have broad application prospects in improving the sensitivity and speed of disease detection, increasing the detection limit and being able to perform multiple analyses and real-time diagnoses [57]. Saliva and gingival crevicular fluids are easily available biological fluids for detecting the emerging biomarkers for the diagnosis of oral diseases in a non-invasive manner. For example, nano-sensors of oral fluids have been developed according to the differences in mRNA/proteins in the saliva of normal populations and patients, which have been used in the diagnoses of some diseases. They can rapidly detect mRNA, miRNA, inflammatory biomarkers and other various protein markers related to oral diseases, and they can also distinguish specific malignant tumor cells with high sensitivity [58,59].

Nanoparticles modified with multi-functional ligands or different antibodies can specifically bind to and accumulate multiple biomarkers, and thus they can simultaneously improve the sensitivities of detection and analysis for multiple disease markers [60,61,62]. Nano-biosensors can quickly identify specific biomarkers and can be used for real-time field detection [63,64]. The development of integrated technology of intelligent nano diagnosis and treatment has far-reaching significance for real-time disease diagnosis and clinical on-demand therapy [65,66,67]. Currently, the gold standard in the diagnosis of periodontitis is through X-ray imaging, observing degrees of alveolar bone resorption. However, it is difficult to predict the occurrence and monitor the recovery of periodontitis in real time. Encouragingly, monitoring gingival crevicular fluids (GCFs) is now regarded as a dependable new strategy for evaluating periodontal tissue status [68,69]. By analyzing Na^+^ content in GCFs, a magnetic nanoparticle sensor based on the magnetic nanoparticle membrane and ionic carrier can detect inorganic substances in various polymer materials to evaluate the periodontal tissue growth status [70]. However, the application potential of this new strategy has been limited due to the cost and synthesis process constraints, and thus the development of more convenient, more economical sensors for potential clinical applications has become a new trend at present.

In the diagnosis and cancer treatment, the recognition of cancer tissues or lesion images is very important. For example, X-ray high-absorption gold nanoparticles (GNPs) can be used as a computed tomography (CT) imaging enhancer for in vivo angiography, since GNPs (polymers and mRNA) or their targeted modification (e.g., with epidermal growth factor receptor (EGFR), human epidermal growth factor receptor 2 (HER2), RGD peptide, folic acid) can accumulate in tumor tissues and enhance image development [40,71,72]. Nanotechnology also uses near-infrared or luminescence quantum dots to detect oral cancer or other oral diseases [40,73,74]. Ankri et al. [75] combined gold nanorods with EGFR and found that gold nanorods spread gradients from tumor tissue to normal epithelium, which can be used as an objective measurement tool to determine tumor margins. The targeting of gold nanorods to oral squamous cell carcinoma (OSCC) was measured by the diffusion reflection method. A recent study showed that light absorption intensity significantly increased in rats with oral cancer when compared with rats without cancer [76], demonstrating that AuNPs-based high imaging contrast and resolution have great potential in determining tumor margins and distinguishing benign and malignant lesions. Thus, emerging nanotechnology has developed rapidly in the areas of oral biomarker enrichment, multiple marker analyses and real-time diagnosis and has broad application potential in oral disease diagnosis and management.

## 5. Disease Treatments and Tissue Engineering Applications

The application of nanomaterial technology has become more and more extensive in the area of oral disease treatment, which involves drug delivery, advanced material construction, tissue engineering correction and restoration, covering dental pulp, dentin, periodontium, implant, orthodontic, maxillofacial surgery and other disciplines. The following sections describe some parts of the applications.

### 5.1. Oral Cancer Treatment

Oral squamous cell carcinoma is one of the common malignant tumors in the head and neck, accounting for about 80% of the maxillofacial malignant tumors. Oral cancer is associated with high incidence and mortality, with a higher proportion in developing countries, which brings a grievous menace to health. According to the occurrence sites, oral cancer can be divided into tongue cancer, gum cancer, buccal mucosa cancer, palate cancer and lip cancer. The most widely used tumor treatment methods are surgery, chemotherapy and radiotherapy. Traditional therapies are often accompanied by some difficult problems of safety and side effects. Tissue trauma and inflammation caused by surgical resection may lead to tumor recurrence and metastasis, while the significant side effects brought by radiotherapy and chemotherapy can affect treatment success and greatly increase the pain of patients during clinical treatment [77,78,79,80].

In recent years, nanocarriers that deliver drugs through nanoplatforms to treat cancer have become a promising alternative therapy. More and more new means and technologies have been used in tumor therapy, including photodynamic therapy, immunotherapy, gene therapy, cell therapy and RNA interference (RNAi) therapy. The application of nanotechnology can effectively avoid the degradation of nanomaterials/drugs during circulation in vivo and precisely deliver nanomaterials/drugs to targeted tumor tissues and cells. These new technologies will thus improve drug bioavailability, reduce biological distribution of nano-drugs in other organs and increase concentrations of drugs accumulated at the lesion locations with a reduced systemic toxicity [81,82].

**Figure 3:**
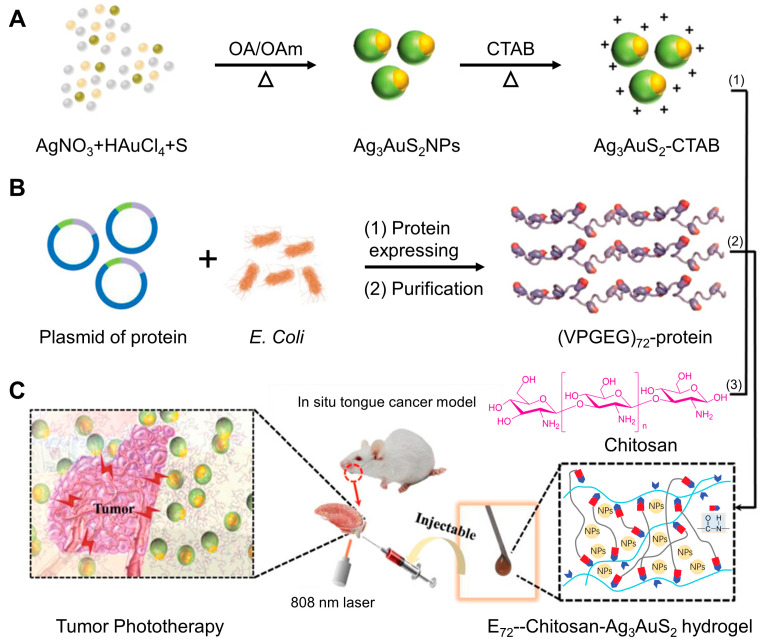
A schematic representation of the preparation of protein hybrid hydrogels for orthotopic tongue tumor treatment. (**A**) Heterogeneous Ag_3_AuS_2_ nanoparticles formed by using the solvothermal method. (**B**) *E. coli* bacteria are induced to produce the negatively charged protein E72, which is purified by a chromatographic column. (**C**) The preparation of homogeneous hydrogel combining E72, chitosan and Ag_3_AuS_2_ NPs through EDC/NHS imide bond formation. Reproduced with the permission, copyright John Wiley and Sons, 2021.

Nanomaterial technology for cancer treatment includes metal nanoparticles, polymer nanoparticles, polymer vesicle/micelle, lipid-based nanoparticles, hydrogels, nanogels, dendrimers, exosomes and so on. Liu et al. [83] prepared a kind of polyethylene glycol-stabilized gold nanoparticle (AuNP) that has a conjugated podoplanin (PDPN) antibody with the cytotoxic drug doxorubicin (DOX). These nanoparticles demonstrated excellent anti-tumor therapeutic efficacy following laser irradiation and can be used as a potential multi-functional platform when combined with chemotherapy/photothermal therapy strategies for oral cancer treatment in vivo and in vitro. Su et al. [84] prepared a new hydrogel that can be injected into orthotopic tumor tissue, and its light-responsive characteristics to near-infrared (NIR) are being utilized for superior photothermal therapy (PTT) for anti-tumor treatment. The photothermal effect of hydrogels is derived from the reticulated structure which consists of negatively charged proteins, chitosan molecules and Ag_3_AuS_2_ NPs. In addition, the flexible hydrogels have natural biocompatibility without posing any toxic side effects on the cells and surrounding biological tissues. These hydrogels can effectively heal orthotopic tongue tumors through one-time photothermal therapy, since the characteristic of slow drug release from the hydrogels can effectually inhibit the possibility of tumor recurrence (Figure 3).

In recent years, immunotherapy by inhibiting checkpoint molecules has become an important part of successful tumor treatment [85]. Studies have shown that, in the diseased tissues of patients with oral cancer, the expression of programmed cell death 1 ligand 1 (PD-L1) is closely related to tumor size, metastasis and other malignant biological behaviors [86,87,88]. Lenouvel et al. [87] made a mouse oral carcinogenicity model and found that anti-PD-1 antibody treatment could significantly control the mouse oral precancerous lesions.

### 5.2. Treatment of Oral Mucosal Diseases

Oral infectious diseases include oral mucositis, oral candidiasis, periodontal abscess, necrotizing gingival stomatitis, oral herpes simplex and oral papilloma. These diseases are characterized by unknown etiology, various clinical manifestations and numerous therapies. Oral mucosal disease is one of the common complications in patients receiving tumor therapy, which has a high incidence [89]. Mucosal protective agents are considered as the first choice for the treatment of oral mucositis, which can protect oral mucosal cells and promote their regeneration. However, there are many problems in the actual development and application of mucosal protectants due to low adhesion, short retention time, poor lubrication ability, low biodegradability, inflammatory effect and so on. Due to the relatively poor protective effect of mucosal protective agents presently, currently available therapeutic agents for oral mucosal diseases still face great challenges. For example, while sucralfate is commonly used clinically to strengthen the protection of oral mucosa, its strong side effects can increase the pain of patients.

The oral mucosal drug delivery system has been widely concerned by the pharmaceutical industry, and there are still problems facing its development, such as weak dissolution of drugs, saliva carrying drugs into the gastrointestinal tract and physiological barriers in the mucosa affecting drug penetration and bioavailability. Traditional biomaterials for oral diseases have now been combined with many new ideas for devising better methods for drug delivery and tissue engineering applications. Due to its nano-scale structure and unique physical and chemical properties, the nano delivery system technology can effectively improve drug solubility, enable its sustained drug release, improve the oral biodistribution potential, increase the drug diffusion rate for crossing the biological barriers and improve the delivery of small molecules, proteins, peptides and nucleic acids in the oral mucosa. At present, a variety of nano-drug delivery technologies (which have been emerging with an increasing trend year by year) has been used for oral mucosa drug delivery, including silver nanoparticles, liposomes, ionizable lipid nanoparticles, nano-emulsifiers, polymer nanoparticles, nano-suspensions, nanofibers and nanogels.

**Figure 4:**
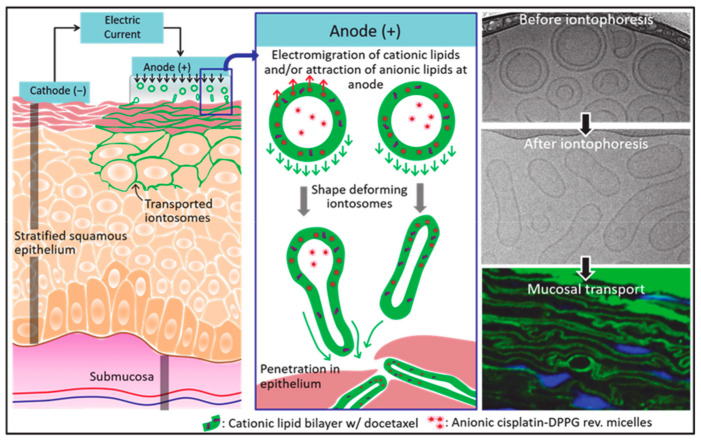
A schematic illustration showing functional properties and iontophoretic mucosal delivery of ionized cationic liposomes. The morphology of ionic cationic liposomes (green) is changed under current stimulation, while anion reverse micelles (red) are attracted to the anode during the process of iontophoretic therapy. The cationic lipid bilayer migrates to the mucosa, and the charge force induces the elongation of ionic liposomes, which is beneficial for the penetration of nanomaterials in the mucosal region through the epithelial cell space. The right image shows the low-temperature TEM and confocal images of ionic cationic liposomes after deposition into mucosa.

The nanoparticle size of lipid nanoparticles facilitates physical adhesion to skin or mucous membranes, which can also be achieved by forming hybrid (nanoparticle/polymer) systems. Sonaje et al. [90] prepared a charged deformable LPS-ionomer, which can be deformed under an electro-osmotic therapy to better penetrate the mucosa to deliver the chemotherapy drugs cisplatin and docetaxel (Figure 4). The combination of ionosomes and electro-assisted delivery provides a new way to solve the problem of the selective delivery of multiple chemotherapeutic drugs to oral mucosa.

Chitosan has been used as a nanomaterial with strong potential for mucosal and engineering applications due to its anti-inflammatory, antimicrobial and free radical scavenging properties. Bilginaylar et al. [91] evaluated the effects of chitosan in preventing oral mucosal diseases caused by the chemotherapy drug methotrexate. The results showed that chitosan application can obviously reduce expression levels of methotrexate-induced tissue inflammation and damage-related molecules including IL-1, TNF-α, MMP-1, MMP-2, caspase-3 and caspase-9, which indicated that chitosan had excellent mucosal protection properties when used alone or as part of a co-delivery drug system.

For oral-mucosal-related diseases, the local drug delivery system enables a highly targeted delivery with a higher drug concentration at the focal site, which can provide a stronger therapeutic effect than systematic delivery. Key benefits of such as delivery system consist of negligible systemic side effects, increased drug delivery efficiency (avoiding waste of drugs in systemic circulation) and enhanced targeted delivery ability with drugs being able to accumulate more easily to the focal site when delivered locally. Choi et al. [92] found, in a beagle model, that a chitosan-coated electrospinning membrane loaded with a low concentration of human growth hormone could quickly repair mucosal ulcers caused by acetic acid smear. The number of promising drug candidates with poor water solubility has been increasing rapidly over the years. For example, Tanja et al. [93] designed an electrospun polycaprolactone (PCL) nanofiber as a nano-drug oral mucosa delivery system for small molecule drugs delivery with a poor water solubility. Compared with polymer films loaded with ibuprofen or carvedilol model drugs, nanofibers effectively improve the efficiency of drug delivery in the oral mucosa.

Overall, with the advancements and improvement of nanomedicine delivery technology and theory, the drug delivery applications in oral mucosal will be more and more extensive, and the problems of oral mucosal nanomaterial technology-based drug delivery system will be overcome one by one, which will accelerate the development and application of related drug preparations.

### 5.3. Treatment of Dental Pulp Disease

Pulp disease refers to a variety of induced conditions caused by dental pulp tissue lesions, including pulpitis, pulp necrosis and pulp degeneration. Traditional pulp treatments include pulp capping, pulp amputation or root canal therapy, depending on the condition of pulp infection and lesion. The nerves and blood vessels in the pulp cavity provide nutrients to the tooth hard tissue and mediate the continuous development of the tooth root. As vitality of the pulp is important for the tooth’s longer service life and better use experience, more and more patients are gradually aware of the necessity of retaining pulp activity [94,95]. New techniques are being developed rapidly, such as pulp and vascular revascularization, apically induced regeneration and pulp and tissue regeneration [96,97].

**Figure 5:**
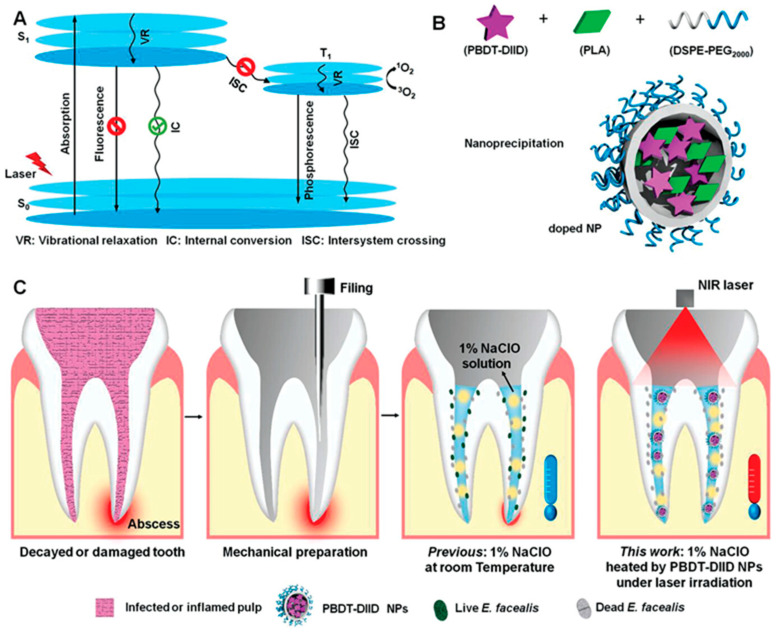
A schematic illustration of semiconducting polymer nanoparticles with intramolecular motion-induced photothermy for tooth root photothermal canal therapy. (**A**) The photophysical processes for the formation of PBDT-DIID. (**B**) The preparation of PBDT-DIID nanoparticles via the precipitation method. (**C**) The heating process of photothermy nanoparticles for root canal therapy by the 1% NaClO solution under 808 nm laser irradiation. Reproduced with the permission, copyright John Wiley and Sons, 2022.

Some nanomaterials, such as gold, silver and polymer nanomedicines, are considered useful disinfectants for root canal therapy due to their broad-spectrum antibacterial and anti-biofilm activities [98]. Realization of these new nanomaterial technologies has profited from the development of advanced materials science. Unexpectedly, nano-photo-sensitizers-based nanomaterials can achieve outstanding antibacterial effect combined with photodynamic therapy. Duan et al. [99] designed and synthesized a novel conjugated polymer nanoparticle with semiconductor properties combined with double fused isoindigo (DIID), and these PBDT-DIID (DIID-based semiconducting conjugated polymer) NPs were found to possess biological safety and outstanding photothermal conversion efficiency, which significantly improved the efficacy of root canal therapy in human root canal infection models (Figure 5). In another study, the root canal sealant was prepared by mixing silver nanoparticles with dimethylaminohexadecyl methacrylate (DMAHDM) polymers, which showed outstanding air tightness, fluidity and antibacterial properties and effectively inhibited the proliferation of pulp biofilm and the recurrence of root canal infection [100]. Nanomaterials have been developed for the treatment of pulp exposure and pulp regeneration. Imura et al. [101] prepared a pulp capping agent, which effectively promoted the differentiation of odontoblast cells and the formation of calcified tissues by utilizing the scaffold properties of nano-scale hydroxyapatite (NHAP) combined with fibroblast growth factor-2. In a rat model, Yoshida et al. [102] tested the combined application of dental adhesive materials (Super-bond (SB)) and NHAP in the treatment of pulp diseases and showed that the nanomedicines induced the formation of dense dentin in the pulp-exposed area.

Due to the complex anatomical structure of dental pulp, root canal therapy is still hugely challenging, and the difficulty of eliminating the biofilm of the root canal wall increases the difficulty of clinical treatment. Dental pulp treatment has been transitioning from cold tooth filling to hot tooth treatments. While the traditional root canal materials have the primary concern of lacking antibacterial properties, the development of nanomaterials has brought many choices and opportunities for oral root canal materials. Newly developed filling drugs (including gold, silver nanoparticles and hydrogels) have strong antibacterial effects and can effectively repair the root canal lateral wall. Dental pulp regeneration is the next target that people are eager to solve. Overall, new dental pulp repair and regeneration technology will alleviate people’s suffering, and the application of nano-biomaterials will give hope to completely kill stealthy dental pulp pathogenic bacteria.

### 5.4. Treatment of Periodontal Disease

Periodontal disease is a complicated disease with complex etiology that destroys collagen and tooth support materials. The main pathological features show a disordered microenvironment under the combined influence of a variety of pathogens and microorganisms, including porphyromonas gingivalis and actinobacillus concomitant [103,104], which lead to the destruction of dental supporting tissues. The local microenvironment of active periodontitis is characterized by an imbalanced immune response, and endogenous inflammatory factors, matrix metalloproteinases and prostaglandins can cause secondary damage to periodontal supporting tissues.

Gum health is damaged in the early stage, and the periodontal ligament and alveolar bone are gradually destroyed in the chronic inflammatory stages [105]. In different populations, the degree and progression of periodontal disease vary from person to person. In terms of clinical manifestations, mild gingivitis is mainly characterized by gingival inflammation and bleeding, while severe gingivitis includes loss of alveolar bone, broken gums and separation of periodontal pockets from the teeth [106,107]. The persistent malignant progression of periodontitis may cause acute systemic diseases as well [108,109], such as respiratory diseases, diabetes, digestive tract diseases, cardiovascular diseases and cancer. About 10 percent of adults suffer from severe periodontal disease, which poses a long-term threat and challenge to human health [110].

The treatment of periodontal disease is based on the delivery of antibacterial agents and host regulators [111,112]. While the destruction of periodontal tissue is traditionally thought to be irreversible, advanced oral nanomaterials have changed this stereotype and are effectively used in the treatment of periodontitis. They have developed rapidly in areas such as periodontal diagnosis (periodontal sensors), antibacterial therapy (including antibacterial/anti-inflammatory materials, periodontal antibacterial drug delivery carriers) and tissue engineering (periodontal treatment scaffold materials). NPs such as Ag and zinc oxide are good antibacterial agents [113,114] which can achieve antibacterial effects by destroying bacterial outer membrane and inner membrane, interfering with bacterial mitochondrial respiratory chain, producing reactive oxygen species, depleting intracellular ATP and inhibiting DNA replication [115]. Since modulating the periodontal local inflammatory microenvironment is thought to be conducive to periodontal healing, Liu et al. [116] integrated polylactic acid/polyethylene glycol co-functionalized MSN into injectable polylactic acid nanofiber sponge microspheres, which were loaded and could locally release anti-inflammatory molecules (miR-10a, IL-2 and TGF-β). When this composite was delivered locally, it was shown to maintain local immune homeostasis and reduce alveolar bone absorption.

### 5.5. Tissue Engineering Applications

Potential applications of tissue engineering in stomatology include maxillofacial fractures, bone augmentation, temporomandibular cartilage regeneration, dental pulp repair, periodontal ligament regeneration and osseointegration [5]. Stem cells are widely used, mainly from dental pulp tissue, periodontal ligaments and alveolar bone. Nanotechnology has improved scaffold materials which can provide unique three-dimensional matrix conditions for cell and tissue growth [117]. In addition, many biological proteins and active molecules are also needed to be combined in tissue engineering to induce stem cell differentiation, control cell behavior and create structures similar to enamel, dentin, dental pulp and alveolar bone [118,119,120].

#### 5.5.1. Application in Oral and Maxillofacial Bone Repair

The vigorous development of tissue engineering technology and regenerative medicine provides new ideas and directions for the regeneration and restoration in oral and maxillofacial bone defects [121,122]. Bone is composed of both organic and inorganic compounds, mainly natural nanocomposites such as collagen and hydroxyapatite. The tissue engineering field has made remarkable achievements in the development of bioactive bone materials or substitutes, which are regarded as 3D scaffolds with certain osteogenic activity, obtained through customized processing technology or functional modification. They have been widely used in bone injury repair applications and have shown good clinical therapeutic effect. The three basic components of bone tissue engineering technology include scaffolds, bioactive molecules and cells [123].

Commonly used bone tissue engineering scaffold materials include metals, bioactive ceramics, natural polymers and synthetic polymers [85,124]. The physical and chemical signals that affect the osteogenic induction activity of bone tissue engineering materials mainly include bioactive ceramics, bioactive ions, material structures and biomolecules. Since the bone substitute materials are fully synthesized and as the particle size decreases and the surface area increases, the scaffold material has the advantages of osteoinduction, non-sintering, good permeability, inability to be degraded by bone resorptive cells osteoclasts, easy shaping and, most importantly, it can stimulate periodontal tissue regeneration. Recently, Shen et al. [125] demonstrated that the slow release of Zn^+^ on the surface of the implanted scaffold endowed the implanted material with good anti-inflammatory effect after the surface of the titanium implant material was modified with a zinc-organic framework, thereby improving the osteogenic induction activity of the implanted material. Pan et al. [126] prepared biomimetic polysaccharide hydrogels, compounded modified nano-hydroxyapatite particles, to obtain a composite scaffold of hydrogel/hydroxyapatite stent, and the volume of mandibular new bone in rats increased by more than 50%, showing the potential to maintain dimensional alveolar ridge.

The process of bone regeneration is finely regulated by various growth factors in time and space [127], which is mainly manifested in two stages: vascularization and osteogenesis. In the process of bone regeneration, cytokines such as bone morphogenetic protein 2 (BMP-2) and vascular endothelial growth factor (VEGF) play key regulatory roles in the mechanism of osteoinductive regeneration and angiogenesis. Liu et al. [128] combined BMP-2 and VEGF to improve the vascularization and osteogenic regeneration of mineralized collagen porous scaffolds. In a rabbit mandibular defect model, the study confirmed that the combined effect of the two factors on the surface of the scaffold synergistically promoted the formation of new bones and blood vessels in the mandible.

#### 5.5.2. Implant Restoration

Implant osseointegration is affected by many important factors, and bone condition, especially alveolar bone quality, is considered the basis of success with the determining factors including surface contact area and damage surface morphology. The success rate of dental implants depends on the quality and volume of the alveolar bone. If the alveolar bone size does not meet the requirements, enlargement is required. The regeneration and restoration of alveolar bone are the primary challenges that need to be solved in the field of oral implantation, and the continuous development of new bioactive materials and tissue engineering technologies provides hope for the high-quality regeneration of alveolar bone.

**Figure 6:**
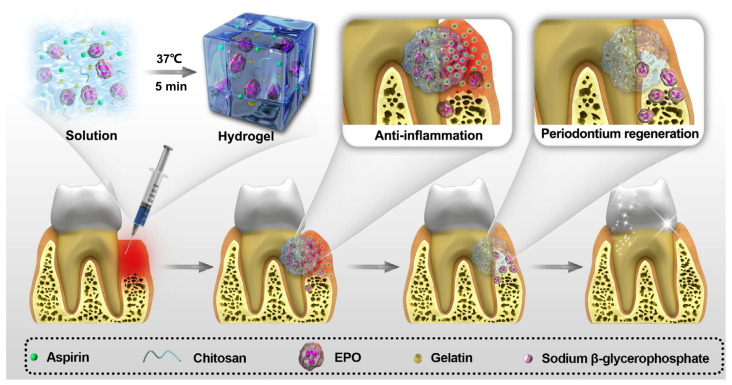
A schematic diagram of an injectable and thermosensitive hydrogel, its preparation and application. A chitosan (CS)/β-glycerophosphate (β-GP)/gelatin hydrogel loaded with aspirin and erythropoietin (EPO) is fabricated at room temperature, which shows no toxicity or any side effects both in vivo and in vitro, exhibiting the ability of promoting periodontal regeneration and controlling inflammation by controlled release of aspirin and EPO. Reproduced with the permission, copyright Elsevier, 2019.

Contrasting with the rough surface of traditional materials, nanomaterial implants have the advantages of having the largest specific surface area, functional group modification and inclusion of certain cytokines or growth factors that are conducive to the adsorption of proteins and adhesion of bone forming cells osteoblasts, which are significant for early implant osseointegration. Bioactive ceramics can improve the bioactivity of implanted scaffolds and have great potential in promoting alveolar bone regeneration. Various coating techniques, such as hydroxyapatite coating, drug coating and metal ceramic coating, can add growth factors, peptides, proteins and nucleic acids drugs (DNA, mRNA and microRNA) to improve the adhesion ability of osteoblasts, induce osteogenic differentiation, promote bone formation around implants to a certain extent and prevent peri-implantitis. Boda et al. [129] added BMP-2 biomimetic polypeptide onto mineralized PLGA, collagen and gelatin composite nanofiber scaffold by ion chelation and found that the modification with BMP-2 effectively promoted the repair of alveolar bone defects. However, there are still many problems that limit the clinical application potential of this strategy, including poor histocompatibility of coating materials, lack of implant binding strength, low fracture resistance and bending toughness and weak weight bearing [71,74]. The inflammatory microenvironment caused by periodontitis or implant material implantation will lead to the occurrence of fibrosis tissue, aggravate the resorption of alveolar bone and lead to the reduction in alveolar bone mass. Thus, in the process of alveolar bone repair, biomaterials are required not only to have some osteogenic activity, but also to regulate the local inflammation and immune microenvironment to guide the formation of new bone. Xu et al. [130] combined the anti-inflammatory aspirin and bioactive erythropoietin into chitosan/β-glycerophosphate/gelatin hydrogels for periodontitis treatment, which was shown to be able to promote alveolar bone regeneration considerably (Figure 6).

#### 5.5.3. Application of 3D Bioprinting Technology in Stomatology

The craniomaxillofacial structure is quite complex in the human body, as it includes various tissues such as ligaments, cartilage, muscles, bones and teeth, as well as supporting structures such as nerves and blood vessels [122]. The defects of oral and craniomaxillofacial tissues will have a huge impact on the patient’s oral function and appearance. While the general method is very challenging for the complete recovery of these tissue injuries, the 3D printing technology, by precisely controlling the composition and spatial distribution of cells and biomaterials, can fabricate scaffolds for promoting tissue repair and regeneration. Bioactive 3D-printed scaffolds, tissue, and organ-analogue biocompatible materials are exciting alternative strategies, and these technologies could overcome parts or most of the crucial challenges in the realm of stomatological diseases [131,132,133,134].

##### Regeneration of Cranial and Maxillofacial Bone and Cartilage

Jaw defects are the most common oral and maxillofacial disorders. At present, the main treatment methods include autologous bone transplantation, allogeneic transplantation and repair with artificial substitute [135]. Although autologous bone transplantation is considered the gold standard for bone defect repair, there are problems such as limited bone mass in the donor site, postoperative bone defect in the donor site and complications. Allograft bone transplantation also faces severe challenges such as immune rejection and infection. However, tissue-engineered bone based on biological scaffold materials and stem cells provides a new way and hope for jaw regeneration [122,134]. Collagen is an important part of human bones, which can enhance its biomechanical properties, bone conduction and bone induction ability [136]. Martin et al. [137] functionalized the 3D-printed polylactic acid–collagen scaffold with minocycline and hydroxyapatite nanoparticles, which could significantly stimulate the adhesion, proliferation and osteogenic gene expression of human mesenchymal stem cells and inhibit the growth of *Staphylococcus aureus*. Liu et al. [138] fabricated silk fibroin/collagen/hydroxyapatite scaffolds using low-temperature 3D printing and loaded recombinant human erythropoietin for alveolar bone defect reconstruction. Dhivya et al. [139] developed injectable thermosensitive chitosan/β-glycerophosphate hydrogel doped with zinc and nano-hydroxyapatite particles and showed that it could promote osteoblast differentiation and regeneration of rat tibial critical bone defects in vitro and in vivo.

The temporomandibular joint is one of the most commonly used joints in the human body, which connects the articular surface of the mandibular condyle and the temporal bone [140]. Trauma or inflammation of the temporomandibular joint can cause temporomandibular joint disorders, resulting in joint pain, defects and mandibular movement disorders [141]. Due to the limited blood supply, the self-repair ability of temporomandibular joint cartilage defects is insufficient. Traditional clinical treatment strategies such as physical therapy, drug therapy and joint puncture are still unable to restore the biological function and mechanical properties of the original cartilage [142]. Tissue engineering technology based on stem cells, biological signal factors and scaffold materials brings hope for structural regeneration and functional recovery of defective cartilage. Heirani-Tabasi et al. [143] cultured adipose mesenchymal stem cells in injectable chitosan/hyaluronic acid hydrogel carrying extracellular vesicles of human articular chondrocytes. In vitro experiments showed that the cartilage differentiation ability of stem cells was significantly enhanced. Collagen has special biological activity and adjustable physical and chemical properties, which can promote cartilage cell (chondrocyte) adhesion, migration and proliferation and has great potential in repairing cartilage defects. Yang et al. [144] mixed collagen with sodium alginate as bio-ink, added primary chondrocytes isolated from the articular cartilage of newborn rats and found that sodium alginate/collagen (SA/COL) bio-ink could promote the proliferation of chondrocytes and the expression of cartilage-specific genes.

##### Periodontal Tissue Regeneration

Periodontal tissue is the supporting structure of teeth, which is composed of gingiva, periodontal ligament, alveolar bone and cementum. It has the function of fixing, supporting teeth and transmitting chewing force [145]. Periodontitis is a plaque-induced chronic infectious disease. Factors such as flora imbalance and autoimmune response will lead to progressive destruction (gum retraction and bone resorption) and loss of attachment of periodontal supporting tissues (gums, alveolar bone, periodontal ligament and cementum), resulting in the loosening and shedding of teeth and oral dysfunction [146,147]. At present, the traditional periodontal treatment strategy is mainly based on anti-infective treatment based on plaque removal. However, the current periodontal surgery and non-surgical treatment for clinical use cannot reconstruct the morphology, structure and function of periodontal tissue, and it is difficult to achieve periodontal tissue remodeling and functional reconstruction.

Technology based on 3D printing has been shown to be able to achieve periodontal tissue regeneration by creating scaffolds with specific shapes and sizes [148]. The discovery of periodontal ligament stem cells provides an important biological basis for periodontal tissue regeneration [149]. Ganguly et al. [150] showed that the sustained and slow release of moxifloxacin hydrochloride could be achieved by using chitosan hydrogel as the carrier, which had significant antibacterial activity against the periodontal pathogens *Actinobacillus actinomycetemcomitans* and *Streptococcus mutans*. Xu et al. [130] used thermosensitive chitosan/β-glycerophosphate/gelatin hydrogels combined with aspirin and erythropoietin to successfully control periodontal inflammation in rats, restore alveolar bone height, bone mineral density and bone volume fraction and achieve complete alveolar bone regeneration. Lin et al. [151] developed a collagen-based biomimetic microfiber system that can withstand functional loads to help periodontal ligament regeneration. Corrugated microfibers of 3D-printed collagen retain the vitality of periodontal ligament cells, showing a trend of promoting healing and regeneration, and are expected to achieve periodontal ligament regeneration.

##### Regeneration of Pulp Nerve and Blood Vessel

Dentin is the hard tissue that constitutes the main body of the tooth, and it is important to maintain the structural and functional integrity of the pulp–dentin complex [152]. While dentin is responsible for protecting the pulp, the pulp is responsible for maintaining the vitality of teeth [153]. The central pulp cavity is filled with pulp tissue, containing a variety of cells and neurovascular structures. Trauma or infection can damage the tooth tissue, and severe cases will lead to pulpitis and periapical lesions. The traditional treatment methods currently used in clinical practice, such as root canal therapy and vital pulpotomy, can repair damaged teeth and eliminate root canal inflammation to a certain extent; however, they cannot restore the integrity of the structure and function of the teeth themselves [154] and cannot replace the biological function of naturally healthy teeth [66].

The use of 3D printing technology can prepare a scaffold with a high-precision three-dimensional structure, provide a suitable microenvironment for cell adhesion and growth and provide the possibility for the regeneration of the pulp–dentin complex [155]. In order to restore the tubular dentin structure and the vascular distribution, innervation and immune function of the pulp, it is expected to become an ideal treatment method. Moreira et al. [156] used chitosan/β-glycerophosphate hydrogels combined with photobiomodulation therapy to significantly improve the activity, proliferation and migration of apical papilla stem cells, which can induce stem cell homing and generate predentin and pulp tissue in the rat molar pulp cavity. El Ashiry et al. [157] used chitosan/sodium alginate hydrogels to load growth factors and canine autologous dental pulp stem cells, which can generate the dental pulp–dentin complex in the pulp cavity of young permanent incisors in dogs, successfully eliminating periodontitis and continuing the development of roots. Yu et al. [158] co-cultured 3D-printed alginate–gelatin scaffold with human dental pulp stem cells and found that it could promote cell adhesion and proliferation, enhance the formation of calcium nodules and up-regulate the expression of mineralization genes, which could effectively promote the osteogenic and odontogenic differentiation of human dental pulp stem cells.

So far, 3D bioprinting has shown great promise for drug discovery and preclinical testing, including the regeneration of cranial and maxillofacial bone and cartilage. However, despite the research already conducted and significant progress achieved in the field of stomatology, there are still many challenges. With rapid advances in engineering, biology, chemistry, computing and medicine, the true potential of 3D bioprinting technology can only be realized in laboratory and clinical settings if stomatology continues to be integrated with other disciplines.

## 6. Safety and Technical Analyses

### 6.1. Safety Analyses

The development of nanotechnology changes with each passing day. While nanoparticles have a large specific surface area and volume ratio and have a high absorption rate in skin, lung, liver, kidney, digestive tract and other tissues/organs, the ideal nanoparticles should be non-toxic, biodegradable, and bio-inert in different tissues. However, there have been more and more clinical concerns about the safety of various biomaterials in recent years.

The delivery of nanoparticles to organs and tissues throughout the body along with blood circulation can cause significant problems. The electrical toxicity of cationic nanomaterials can cause irritant responses in the biological and physical barrier stage of systemic circulation, including alveolar cell damage that may induce acute respiratory system inflammation and tissue damage. As metal nanoparticles generally cannot be completely metabolized, their retention in organs and tissues will result in long-term toxicity. In addition, polymer metabolism will increase the burden of the kidney and liver and may cause cardiovascular and other organ injury [71,72]. Furthermore, this situation can lead to potential long-term biological toxicity, which is difficult to monitor and evaluate continuously.

The local application of nanomaterials will interact with the environment, which may lead to adverse reactions in immune systems. For example, metallic element nanomaterials with high molecular weight are often selected for medical applications. Due to the unique physical and chemical properties of nanoparticles, the body’s immune cells have some preferential effects on them, which may affect their therapeutic effects. Apart from the implant immunogenicity issue, the failure of implant surgery is usually due to the bacterial infection of the surrounding tissue, which puts forward higher requirements for the antibacterial performance and biosafety of antibacterial agents.

Tissue engineering composite materials (for tooth loss management and bone defect repair) also have faced many problems, including limited stem cell sources, large cell demand, easy contamination and difficulty in inducing cytokines/growth factors in vitro. In bone regeneration treatment, due to the limited source of autologous bone, the use of allogeneic bone brings in problems such as immune rejection and disease cross contamination. New materials such as bioactive 3D scaffolds also face the problems of immune rejection and low bioactivity. Genotoxicity and ethical issues also must be considered in the field of biosafety. Gene therapy, such as gene correction and gene editing, may lead to irreversible changes in genetic materials (chromosomes and genes). The direct contact with blood, skin and mucous membranes of nanomaterials may also induce genetic toxicity.

To avoid being harmful to human health, tissue engineering materials for human use demand absolute safety, no cationic toxicity and no immediate and long-term immunogenicity. Tissue engineering has put forward better requirements for green materials, biocompatible materials and long-term stability materials.

### 6.2. Technical Difficulties Limit Further Developments

With the development of nanotechnology, people put forward higher requirements for the manufacture and technology of nanomaterials, and the design concept and synthetic pathways are gradually shifting to clinical needs. For example, material morphology, optimal diameter, targeted modification, small molecule drug or gene drug selection are chosen according to different uses of organs and nanomaterials. Considering the complexity of the oral cavity and maxillofacial region, it is necessary to design appropriate physical and chemical properties of nanomaterials according to the oral and maxillofacial microenvironments. In oral disease treatment, the dynamic load measurement, fatigue test and biosafety of metal, ceramic, polymer and other prosthetics still need further clinical research. While bio-nanomaterials have good tolerance, further research is needed in the aspects of their tracing in vivo, slow release, concentration control, accurate evaluation and organism interaction. These technical difficulties have limited the development of oral nanomedicine to some extent [40,159].

## 7. Discussions and Problems Faced

Multi-functional oral nanomaterials have been designed for potential clinical applications by systematic drug delivery or local delivery that faces the special complex oral microenvironment. They must have biocompatibility and stable long-term physicochemical properties. In order to enhance the therapeutic effect of antibacterial agents, regenerative bioactive substances and immune therapeutic agents, oral nanomaterials are required to have protective properties, improve targeting and penetration abilities of biological and physical barriers in oral and maxillofacial microenvironments, achieve excellent accumulation in lesion sites, maintain effective nanomedicine concentration and control the sustained release of drugs. Here, we have summarized applications and functional materials of nanomaterials and tissue engineering for oral diseases (Table 1).

Oral nanomaterials have excellent dispersibility and high specific surface areas and volume ratios, which are conducive to active drug loading. Targeting pathogenic bacteria or inflammatory cells, they are widely used to treat oral infectious diseases such as dental caries and periodontal disease. Combined with physical technologies, such as sonodynamic, photodynamic and photothermal therapies, nanomaterials can exert an enhanced therapeutic effect for targeted treatment in organs or tissues that are difficult to reach by conventional drug delivery systems, such as dental pulp diseases and periosteal infections. Excellent oral nanomaterials should be able to continuously and stably release active drugs to achieve therapeutic effects without affecting the homeostasis of oral flora and destroying the oral microenvironment, such that they can effectively eliminate root canal biofilm, improve implant tissue binding ability and treat dental pulp diseases. Tissue engineering materials, hydrogels, active skeletons, nanofibers and nano-films have strict requirements for biocompatibility, long-term stability, antibacterial activity and osteoinduction ability. Hydrogels and bioactive scaffolds can be used for cell and tissue regeneration in periodontal, dental pulp and maxillofacial bone defects. The development of nanomaterials with good oral mucosal adhesion, such as nano-films and composite hydrogel materials, can be used for the treatment of mucosal diseases. In addition, the usage of nanomaterials may not be enough to solve all the complex diseases. Combined treatment and co-delivering among nanomaterials, therapeutic agents, genes and some other chemical drugs (e.g., chemotherapeutics) have become robust approaches to gain synergetic therapeutic effects. Moreover, the designability, versatility and drug delivery capabilities of nanomaterials can also play a synergistic therapeutic effect to deal with the intractable cases effectively.

However, further work is required for developing clinical demand-oriented nano-drug delivery systems. It is gratifying that we achieved some clinical application results currently. For example, nano-HAP, calcium phosphate (CAP) nanomaterials have a similar chemical structure to hard human tissues such as bone and teeth, and they can be used as effective delivery carriers for various therapeutic agents or biomolecules, including nucleic acids, proteins, peptides, antibodies and chemical drugs [160,161]. Nano-HAP, CAP-based nanomaterials were widely used in the clinical treatment of oral diseases due to its osteogenic properties and biocompatibility [160,162], and it was applied in tissue remineralization [163,164], dentin hypersensitivity reduction [165,166], orthodontics treatment [167], transplantation materials application [168,169] and periodontal defect therapy [170], which have a great potential in dentistry with a broad application prospect. Demineralized bone matrix (DBM) is an allograft that contains a large amount of osteogenic protein components, little calcium-based solids, inorganic salts and trace cell fragments without mineral components [171,172], which can be used as bone conduction and bone induction biomaterials because of its excellent biocompatibility [173]. It was approved to be used for the healing of bone defects and spinal fusion. DBM products have various forms in clinical practices, such as sponges, strips, injectable putty, paste, etc. [174], and all of these forms obtained satisfactory therapeutic effects. The biocompatibility and versatility of nano-HAP, CAP and demineralized bone matrix (DBM) encourage further clinical research to explore its clinical usages.

However, serious challenges remain to be solved for developing clinical demand. The long-term inhibitory effect of drug-resistant bacteria lacks a clear theoretical basis. The unclear and complex ecological environment of the oral cavity brings challenges to the elucidation of molecular mechanisms in antibacterial, anti-inflammatory and anti-viral therapies. Further animal experiments and in vivo studies are required to investigate the functionality and long-term biocompatibility of composite nanomaterials incorporating multiple drug delivery systems. Further work is required to study how nanomaterials interact with the complex local microenvironment and the immune system in vivo. Furthermore, the anatomical complexity of oral and maxillofacial areas requires the design of appropriate physicochemical properties of nanomaterials according to the actual microenvironment. In considering how to organically combine different nanomaterials with drug delivery systems, researchers should also consider how to improve the druggability and long-term biological safety.

## 8. Conclusions and Future Prospects

Nanomedicine delivery carriers and tissue engineering materials for oral medicine have obtained promising results due to their unique physical and chemical properties and biological advantages. The design ability, antibacterial activity, biocompatibility, photothermal sensitivity and electrical conductivity of nanomaterials offer promising application prospects in the medical field. Moreover, the addition of traditional materials to the nanomaterial systems forming some novel nanocomposites can further improve the biological, chemical and mechanical properties of the materials. With the development of cell biology, molecular biology, materials science, nanotechnology and stem cells, the research, development and clinical applications of new nanomaterials are conducive to promoting the reform and innovation of oral materials. Encouragingly, due to their small particle size, high antibacterial activity, biocompatibility and biodegradability, nanomaterials are now finding their ways to provide solutions for tissue engineering for the development of oral tissue rehabilitation and regeneration technology.

However, nanomaterials for oral medicine still face challenges in design concepts and synthesis, such as difficult processing and relatively high costs. The sustained release control, precise evaluation, organism interaction and long-term stability of these nanomedicines in drug delivery systems still lack detailed studies, which has become an obstacle for their clinical applications. How to make better use of the characteristics of nanomaterials and reduce the cost while improving the therapeutic effects is still a direction that needs to be explored. Nanotechnology has been widely used in various medical and oral materials. The applications of novel advanced nanomaterials in the field of stomatology have great significance to the modification and innovation of existing materials for oral medicine. In the near future, nanomaterials will be further explored and studied so as to construct greener and safer nanomaterials with promising clinical application prospects, bringing a better tomorrow to oral medicine.

## Figures and Tables

**Table 1:** Applications and functional materials of nanomaterials and tissue engineering for oral diseases.

Disease Intervention Types	Regulation Types		Nanomaterial Types (Part)	Materials (Part)	Refs.
Nanomaterials in prevention of oral diseases	Prevention of bacterial growth		Metals, polymers, hybrid nanomaterials, nanocomposites	Au, Ag, Zn NPs, ZnG, chitosan, dextran	[35,37,40]
	Demineralization prevention and tissue mineralization promotion		inorganic systems, nanogels, hybrid nanomaterials	Ag, CaF_2_, NACP, Si/Ca/SrNPs	[43,46]
Diagnosis of oral diseases			Polymers, inorganic systems, nanocomposites, quantum dots	Au, Fe_3_O_4_ CdTe, ZnS, CdSe	[58,59,63,68]
Oral disease treatments	Oral cancer treatment		Metal/polymer NPs, vesicle/micelle, lipid-based NPs, hydrogels, nanogels, dendrimers, exosomes	Au, Ag, silica, Fe_3_O_4_, PLGA, PLL, dextran, chitosan, liposomes	[81,83,84,86]
	Treatment of oral mucosal diseases		Metal/polymer NPs, ionizable lipid NPs, nano-emulsifiers, suspensions, nanofibers, nanogels	liposomes, PLGA, PNIPAM, dextran, PCL	[90,91,93]
	Treatment of dental pulp disease		Metal/polymer NPs, hybrid nanomaterials, nanofibers, nanogels	Au, Ag, NACP	[95,96,98,99]
	Treatment of periodontal disease		Metals, polymers, hybrid nanomaterials, nanocomposites	Ag, Zn^+^, PLA, PEG, PLGA	[103,107,112,114]
Tissue engineering applications	Application in oral and maxillofacial bone repair		Metals, bioactive ceramics, natural polymers, synthetic polymers, hydrogels, nanogels, nanofibers, hybrid nanomaterials, nanocomposites	HA, collagen, PLGA, chitosan, gelatin, hydroxyapatite	[117,120,121,129,130]
	Implant restoration				
	3D printing technology	Regeneration of cranial and maxillofacial bone and cartilage	3D-printed scaffolds, tissue/organ analogue materials, polylactic acid–collagen scaffolds	Polylactic acid, collagen, chitosan, HA	[132,133,137,138,148,150,152,155]
		Periodontal tissue regeneration			
		Regeneration of pulp nerve and blood vessel			

## Data Availability

Not applicable.
